# Incorporating Online and In-person Book Clubs into Sociology Courses

**DOI:** 10.1177/0092055X18777564

**Published:** 2018-07

**Authors:** Amanda Wyant, Sarah Bowen

**Affiliations:** 1 North Carolina State University, Raleigh, NC, USA

**Keywords:** student learning, active learning, book clubs, class-based exercises, reading, higher-level thinking

## Abstract

Previous research has examined the use of nontraditional readings, particularly fiction, as a tool for teaching sociological concepts. Few studies have specifically looked at nonfiction monographs and ethnographies. This paper extends prior research by exploring how in-person and online book clubs using nonfiction texts can be used as a tool to engage and introduce students to sociological ideas. Book clubs were implemented in eight different sections across three courses. The structure and format of the book clubs varied considerably. We identify best practices for incorporating book clubs into sociology courses. Drawing on data from instructor-designed surveys, institutional course evaluations, and course exams, we also examine how book clubs influence student attitudes and learning outcomes. We conclude that book clubs can be adapted to fit a variety of courses and across different types of institutions.

Previous research has examined the use of nontraditional readings, including fiction and journalistic accounts, as a tool for teaching sociological concepts (Carlin 2010; Castellano, DeAngelis, and Clark-Ibáñez 2008; Clowers and Mori 1977; Hartman 2005). The literature suggests that nontraditional readings can help students overcome their resistance to sociological theory and intimidation over the abstract nature of many standard sociological texts (Castellano et al. 2008; Hartman 2005). Scholars argue that novels and other nontraditional texts provide a “shared reality” within which students can grapple with and discuss sociological concepts in a common context (Castellano et al. 2008; Hartman 2005). Nontraditional texts also engage students by introducing them to other cultures and contexts. Furthermore, Roberts and Roberts (2008) find that when combined with extensive instructor guidance, nontraditional texts may facilitate “deep reading,” or “reading for long-term retention of the material and for comprehension at a level that can be perspective-transforming” (Roberts and Roberts 2008:125). They note that although deep reading is an important sociological skill, many sociology courses allow students to engage in minimalist or surface reading.

In this article, we explore the use of in-person and online book clubs in sociology courses. Our research is unique in two ways. First, it explores best practices for integrating nonfiction monographs, including those targeted to both academic and general audiences, into sociology courses. Previous studies have looked at how fiction can be used to introduce students to sociological ideas (Carlin 2010; Cosbey 1997; Hartman 2005). Fewer studies have focused on using ethnographies or monographs (Castellano et al. 2008).

Second, with several exceptions (Castellano et al. 2008; Jones 1975; Sullivan 1982), most previous research has not examined the effects of nontraditional readings on student attitudes or learning outcomes. In this article, we draw on student surveys and exams to explore how book clubs shape student attitudes and learning outcomes. As such, this study contributes to work by Castellano and colleagues (2008), who argue that nontraditional texts can be used to promote student engagement, increase understanding of sociological ideas, and improve students’ analytical abilities. They compared strategies that incorporated three different types of nontraditional readings (journalistic nonfiction, popular fiction, and mysteries). They found that students were highly engaged with nontraditional readings and felt that nontraditional readings helped them better understand sociological ideas and conduct sociological analyses by illuminating how sociological ideas related to “real life.” Castellano et al. (2008) conclude that nontraditional readings can be successfully incorporated into sociological courses but caution that this requires forethought and that instructors should provide students with structured guidelines. Similarly, Lewis (2004) integrated book clubs into a course on the sociology of mental illness. Lewis finds that the book clubs foster an academic setting that can lead to greater student participation, a higher level of group intimacy, and a sense of student empowerment in their own learning.

In this article, we describe how we integrated book clubs into three different types of sociology courses. We then present data on student attitudes and learning outcomes related to the book clubs. We conclude by making recommendations for how to best incorporate book clubs into sociology courses.

## Integrating Book Clubs into Sociology Courses

### Setting

Book clubs were integrated into eight different sections across three courses at a large public university. Class sizes were relatively small, ranging from 14 to 44 students, with a median class size of 28 students. Book clubs were adopted in a variety of different types of sociology courses: a general education introductory course (Principles of Sociology), a topic-specific introductory course (Jobs and Work), and a topic-specific upper-level course (International Development). Within the introductory courses, the book clubs were implemented in several sections that were specifically reserved for first-year college students and one standard section. The two introductory courses were Principles of Sociology, which focuses broadly on stratification and covers topics such as race, class, and gender and how they relate to socialization, education, work, and family, and Jobs and Work, which focuses on how inequality structures aspects of employment, including occupational sorting, worker control, and technological shifts. International Development examines global patterns of development and the impact of globalization on social and economic change in different parts of the world, with a focus on contemporary issues (e.g., migration and the illicit economy).

Book clubs were used as a tool to provide a shared context for the sociological lessons and debates that were discussed in class. In all courses, book clubs were worth 10 percent of the total grade and included both individual and group components, as discussed below. There was variation in how the book clubs were designed and evaluated across courses.

### Assignment of Books

In each course, students were divided into “book club groups” at the beginning of the semester. Each group read a different nonfiction book related to the course topic (see Appendix for a list of books used). In all courses, the criteria for choosing books were the same. Instructors selected books that were sociologically relevant, related to the course topic, and written in a way that would engage a wide audience. Sociological monographs (Bank Muñoz 2008; Parreñas 2001; Williams 2006) and creative nonfiction or memoirs (Kidder 2003; Nazario 2006; Yousafzai and Lamb 2013) were used. Each course incorporated books from only one category (either sociological monograph *or* creative nonfiction), as opposed to offering books from both categories.

Students were assigned to their book club groups during the first two weeks of the semester. On the first or second day, the instructor summarized the main topic of each book. Students generally had between three and six books from which to choose, although students in one course (International Development, Spring 2009) chose from eight books. After hearing the summaries, students ranked their first, second, and third choices. Book club group assignments were made by the instructor. Students were told they could be assigned to any book from the three they listed; in all cases, instructors were able to offer all students their first or second choice. If it became difficult to offer all students one of their choices, we would recommend making one group larger than the others (to allow for additional students) rather than making a student read a book that they had not requested.

Instructors aimed to offer a sufficient number of books so that students would have a range of options to choose from. However, offering too many books made it difficult for the instructor and the teaching assistant to read all of the books. In some courses, a longer list of book choices was presented to students, and books that did not generate sufficient interest were eliminated. Offering between four and six books seemed to work best.

In all courses, books were chosen to represent diverse topics and perspectives. For example, in International Development, students chose from books focusing on issues such as gender, family, migration, health, and international aid. In Jobs and Work, students chose from books covering a range of occupations (professional work, service work, manual labor). In Introduction to Sociology, books focused on a few main themes from the course: education, work, and deviance. Instructors generally focused on offering books that covered a wide variety of substantive topics rather than choosing books that represented several different perspectives on key debates. However, it would also be possible to organize the book clubs in this way (e.g., by having students read books with competing perspectives on international aid, migration, or the U.S. educational system).

The sizes of the book clubs varied, generally ranging from five to nine students per group in the International Development courses and three to five students per group in the other courses. In one case (International Development, Spring 2015), groups were even larger (11–12 students). Some students said that the size of these groups made working together “a bit hectic” and students were not able to get to know each other as well; the instructor concluded that such large groups did not work well. Group sizes were smaller in other cases.

Instructors found that groups of four to five students worked best. Groups of this size were small enough that all students needed to take an active role. However, they were large enough that tasks could be divided between group members so that students did not become overwhelmed. The only drawback of groups of this size was that because they were relatively small, it could become problematic if students withdrew from the class.

### Structure of Book Clubs

In all cases, the groups met several times throughout the semester to discuss the books. These discussions took place in person and/or via virtual discussion forums. In all cases, instructors assigned guided reading questions in advance, since previous research suggests that reflection questions or prompts help students engage in deep reading (Parrott and Cherry 2011). As discussed in the following, students surveyed about the book clubs said that the guided reading questions were essential to the success of the book clubs.

In all cases, students submitted answers to guided reading questions via online discussion boards. In the International Development courses, each group participated in one or two extended online discussions of the book. In cases where there were two discussions, the first discussion covered roughly the first half of the book, with specific page numbers given, and the second covered the rest of the book. Each online discussion consisted of three deadlines, designed to prompt interaction over a longer period of time (approximately one week). Discussions took place in a Moodle forum, our learning management system, and included both reading questions and guidelines to ensure engagement and interaction between students. On the first day of each discussion, students were asked to answer one of two specific questions. Questions generally required students to cite specific examples or passages from the books in their responses and were also connected to debates or concepts from the course. For example, one prompt, for a book about a 17-year-old’s experience migrating from Honduras to the United States, asked: “How has *Enrique’s Journey* changed your perceptions of the causes or consequences of migration? Name at least two ways, citing specific details or evidence from the book.” The second deadline came a few days after the first, and the third came a few days after that. For these, students were instructed to post replies to other group members’ comments. In their responses, students were asked to disagree, modify, or add to at least two group members’ comments; mention the students by name; and bring in at least one new detail from the book. All three deadlines were part of the same discussion. Students received individual grades for their discussion postings. The specific guidelines helped facilitate interaction between different students within groups and encourage higher-quality comments; however, there was variation in the level and quality of engagement. Students surveyed about the book clubs had mixed feelings about the format of the discussion boards; some said that they would have preferred only one deadline per discussion, but several students requested having “shorter, more frequent discussions.” Some students said that they liked the online format, while others stated that they thought face-to-face contact would have facilitated better discussions.

In the other two courses, students participated in both online and in-person discussions. First, groups participated in four online Moodle forums. The forums were due the night before each in-person session. In the forums, students posted responses to specific guided reading questions designed to assess students’ comprehension of the reading. This was the only individual-writing portion of the project, and students received individual grades for their responses. Each of the four online forums had a different objective. For example, the first discussion focused on understanding the research design and the primary theoretical concepts in the book. Subsequent discussions focused on linkages between the book and course concepts. Each set of guided reading questions concluded by asking students to provide a quote or fact they found interesting and a discussion question. This allowed students to demonstrate their own creative thoughts; the students used these as starting points for the in-person meetings that took place the day after the online forums were due.

In-person meetings gave students an opportunity to discuss and clarify their responses and seek direct feedback from other group members. Group discussions focused on relating course concepts to the group’s book and required students to think more abstractly (e.g., by applying or evaluating rather than recalling or paraphrasing; Geertsen 2003). Students spent a full class period, typically 75 minutes, working on these questions. Students were assigned roles (e.g., leader, scribe, timekeeper) on a rotating basis to ensure that responsibilities were distributed among group members. Students received a group grade for these questions (25% of the total book club grade for the course). Students who were absent the day of the in-class meetings did not receive points for the activity. These “book club working days” almost always had 100 percent attendance.

### Incorporation of Content into Lectures

Instructors incorporated content from books and discussions into course lectures. They used a variety of strategies to do this. For example, in International Development, during a class discussion about the pros and cons of international aid, the instructor asked students who had read a memoir of an aid worker to relay the lessons from the book. As another example, in class discussions for Jobs and Work, the instructor used charts that included examples from the book clubs. During a discussion of sociological research methods, each book club group gave an informal presentation on their books, and the class collectively integrated this information into a chart. The chart included methods-specific categories (e.g., research sample, methods of data collection) and general categories (e.g., key definitions, findings).

### Group Presentations

In most courses, students gave presentations on their books to the rest of the class. The goal of the presentations was to provide an additional means of ensuring that group members understood the materials, as well as reinforce course concepts across the entire class. The structure of the presentation assignments varied. For example, during two sections of the International Development course, students prepared a “virtual lesson” about their book for their classmates and led an interactive, online discussion with the rest of the class on a topic related to the book. Each group prepared materials that conveyed key lessons from the book to the class (e.g., a PowerPoint, Prezi, or video), posted them to the class website, determined an open-ended question to generate discussion among their classmates, and then moderated the online discussion that followed. (All students in the class were required to review the posted materials and then contribute to the online discussion.) Students received a group grade for the presentations (60 percent of total book club grade; the remaining 40 percent, pertaining to the discussion questions, was individual), and the rest of the class received a participation grade for contributing to the online discussion. Although the other students in the class generally found these presentations to be interesting, the quality of the presentations varied between groups.

In Jobs and Work, students gave 15-minute oral presentations summarizing their books. These took place toward the end of the semester. Students had time to work on the oral presentations during the in-class discussions of the books, and they received a group grade for their presentations. During and immediately after the presentations, classmates asked questions about the books to clarify their understanding of the topic, setting, and relationship between the book and course concepts. During one semester of Jobs and Work, each book club group also led approximately half of a 75-minute class session, relating their books to specific course concepts. For example, a group that had read a book about lawyers gave a presentation regarding professional jobs. These content-specific presentations did not work well because students felt that they overlapped too much with the general (summary) presentations; they were only used during one semester.

## Evaluating Book Clubs’ Effectiveness

With the book clubs, we aimed to (1) promote student engagement and (2) increase student understanding of sociological concepts. In this section, we present the results of several evaluation tools designed to assess the effectiveness of the book clubs. Because these book clubs were used in a variety of courses and over a relatively long period of time, we do not have consistent evaluation data across courses. However, our data suggest that the book clubs were successful at achieving these objectives.

### Promoting Student Engagement

The first objective of the book clubs was to promote student engagement. We draw on two sources of data in our assessment of the book clubs’ impact on student engagement: student evaluations (completed in all classes) and student surveys (completed in two classes).

First, students in all courses completed course evaluations. These evaluations followed the standard university format and did not ask specifically about the book clubs. However, we reviewed the responses to the open-ended questions on the evaluations to assess students’ qualitative impressions of the book clubs. In general, course evaluation scores were high. The majority of courses had scores that were at or above the departmental average, although this is consistent with the instructors’ course evaluations more generally. Most of the comments on the evaluations focused on general aspects related to the course or instructor, not the book clubs specifically. Of the comments that did pertain to the book clubs, most were positive. Two students said that they would have preferred just to write a paper on the book instead of engaging in a group discussion, but comments were generally positive. Students said they “loved the book club choices” (International Development, Fall 2009); one student described the books as “not only relevant to the course, but great books” (International Development, Fall 2009). Students expressed a desire to further incorporate the book clubs into class discussions. One suggested that “if we can get other classmates involved, people reading the book can learn even more from other people’s questions, answers, and views” (Jobs and Work, Fall 2014). Students also stated that they appreciated the book clubs as one of a variety of different teaching tools and strategies. For example, one student, who described the class as “engaging,” said that they appreciated “the videos, the book clubs, and charts in class as good structural aids to help with learning” (International Development, Fall 2015). Another student, who evaluated the class as “excellent,” noted that the book clubs and other in-class activities (e.g., films and debates) helped them “learn a great deal about the history of international development and how it relates to current events” (International Development, Fall 2009).

Although students did not state that the book clubs were superior to or more engaging than other class activities, we do not see this as reflecting a weakness of the book clubs. Instead, we view the book clubs as a central part of a portfolio of different strategies for engaging students. Students emphasized that this was a strength of the courses. For example, one student said they “really enjoyed how there was a differentiation of exercises throughout the semester” (International Development, Fall 2010). Another said that they appreciated how the various types of assignments, including book clubs, debates, and tests, “covered a broad range of topics and allowed for everyone to do well” (International Development, Fall 2010).

In addition to the general course evaluations, students in two classes answered specific survey questions about the book clubs. Students in one section of International Development (Fall 2009) filled out a survey midway through the semester, soon after completing the book club assignment. Students in one section of Jobs and Work (Fall 2014) answered questions about the book clubs on an instructor-designed final review. Responses to both surveys were anonymous, and response rates ranged from 74 percent to 82 percent.^1^

In the surveys, students selected a number on a Likert scale ranging from 1 (strongly disagree) to 5 (strongly agree) to indicate the degree to which they agreed or disagreed with a series of statements. Tables 1 and 2 present the wording of the statements, mean scores, and standard deviations for both surveys.

**Table 1.** Student Survey: Perceptions of Book Clubs in International Development Class, Fall 2009 (n = 31).

	Mean Score (1–5)	Standard Deviation
I enjoyed reading the book.	4.5	.7
I enjoyed participating in the online “book club” discussions.	3.3	.9
The book club assignment stimulated my interest in international development.	4.1	.8
The book club assignment helped me apply the conceptual framework in class to real-world events.	4.0	.8

**Table 2.** Student Survey: Perceptions of Book Clubs in Jobs and Work Class, Fall 2014 (n = 14).

	Mean score (1–5)	Standard Deviation
I think book club is a good way to apply course concepts.	3.8	1.1
I think that the guiding reading questions and group discussion questions help make book clubs successful.	4.3	.8
The number of students in book club groups was appropriate.	4.4	1.0
I enjoyed presenting the mini-presentation	2.9	1.4
As an observer, the mini-presentation helped me understand the material for class that day.	3.0	1.2
I think you should do the mini-presentations next semester.	2.8	1.4

Survey questions varied by course, so they are not directly comparable. However, in general, students agreed or strongly agreed with all questions about the value of the book clubs. For the survey conducted in International Development, the highest scores were for the two questions related to student engagement (“I enjoyed reading the book” and “The book club assignment stimulated my interest in international development”). For the survey conducted in Jobs and Work, scores were high for both the question about the book clubs in general (“I think book club is a good way to apply course concepts”) and the guided reading questions specifically (“I think that the guiding reading questions and group discussion question help make book clubs successful”).

Both surveys included an open-ended question that asked students to comment on what they liked about the book clubs or felt could be improved. Echoing the findings from the quantitative analysis, positive comments focused primarily on students’ engagement with the books’ content and the books’ ability to help students apply course concepts to real-world examples. Students said that the books gave them the opportunity to “take a closer look” at key issues through “in-depth, real-world examples” (International Development, Fall 2009). They expressed how the books provided concrete examples of the course concepts (Jobs and Work, Fall 2014). For example, one student said, “I really enjoyed the book club because it allowed me to get a realistic understanding of how everything we have learned played a role in society” (Jobs and Work, Fall 2014). Students also emphasized that they liked how the books allowed them to see how abstract concepts (e.g., globalization or development) played out in the lives of people and communities. One student wrote, “I like that the book club selections are true stories/memoirs, because they provide a real context to what we are learning in the class” (International Development, Fall 2009). Another student wrote, “I really liked how it gave us the opportunity to take a closer, more personal look at some of the repercussions of international development, not just on a general scale like in much of the class discussion” (International Development, Fall 2009). Overall, student evaluations indicate that the book clubs fostered a sense of engagement among students and allowed them to link abstract concepts from the textbook or class discussions to more vivid and concrete examples and stories.

The book clubs were engaging not just due to the content of the books, but also because of the way they fostered sharing and communication between students. Some students mentioned that they appreciated the opportunity to connect with and get feedback from other students. For example, one student wrote, “It was an interesting way to get to know some classmates. That was my main benefit from it, along with the fact that I enjoyed the book.” (Jobs and Work, Fall 2014). Another wrote, “I think that the thing that I liked was how members of my group shared their ideas about my response” (International Development, Fall 2009).

Compared to assigning students to read and complete individual assignments about nontraditional texts, we argue that book clubs have the potential to generate additional benefits in terms of engagement. By requiring that students convey their ideas and teach aspects of the book to others, book clubs can foster students’ sense of ownership of their knowledge and responsibility to share their knowledge with other students. Another big difference is that the book clubs require students to create something—a presentation, video, or class activity—in a collaborative manner. This format puts more responsibility on students to become experts on their subject matter. Finally, by incorporating a variety of questions and assignments, the book clubs provide a setting for students to engage in higher-level thinking, defined by Geertsen (2003) as incorporating both critical and reflective elements, including critical judging, contextualizing, and problem solving.^2^

### Increasing Student Understanding of Sociological Concepts

In addition to engaging students, book clubs helped improve students’ understanding of sociological concepts. The concepts our students examined included the feminization of migration (in International Development) and alienation of workers (in Jobs and Work). The results of one survey, conducted in International Development, suggest that students felt that the book clubs had improved their ability to apply course concepts. One question asked students whether the book clubs had helped them “apply the conceptual framework in class to real-world events.” Although the average score for this question was slightly lower than the scores for the engagement questions (4.0 on a 5-point scale, compared to 4.1 and 4.5), it was still quite high.

In some semesters of Jobs and Work, exam questions were used to directly assess students’ understanding of materials from the book clubs. On one midterm (Spring 2015), students were asked to match sociological terms with their definitions; these terms had been presented by the book club groups. Average scores for this section were high (8.7 out of 10, SD = 1.56, n = 29), which suggests that the book club groups were successful in expressing the meaning of the terms to their classmates and that their classmates retained this information.

Two final exams for Jobs and Work (Spring 2015 and Fall 2015) incorporated questions pertaining to the book clubs. Students were asked to answer three out of four essay questions. Three of these questions focused on the book clubs, which meant that all students answered at least two questions about the book clubs. In one question, students were asked to use examples from the books to evaluate and characterize different types of work situations in terms of their level of alienation. Another question asked students to compare and contrast how the sociological imagination came into play in two books, and a third asked students to analyze how inequality (related to race, class, gender, or ethnicity) was related to at least one book. Average scores were 13.5, 11.7, and 13.8 points out of 15 points, respectively. Despite variation in the scores, they were all relatively high, suggesting that students were able to use concrete examples from the books in discussions of theoretical concepts from the course.^3^

Finally, one final in Jobs and Work (Fall 2015) included 10 multiple-choice questions that assessed understanding and learning based on the groups’ final presentations. Each book club group submitted two questions for the final, and students were required to answer all 10 questions. Four of the 10 questions were answered correctly by all students. For the other questions, the percentage of correct answers ranged from 65 percent to 90 percent (n = 19).

Overall, our analysis of one survey and several exam results suggests that the book clubs contributed to improvements in student learning. Students successfully retained information about their own books while also learning about other groups’ books. Evaluation tools were not consistent across courses and are not directly comparable. Future research can evaluate the impact of the book clubs on student learning outcomes more directly—for example, by comparing student outcomes in two sections of the same course, one with the book clubs and one without.

## Best Practices for Book Clubs

The structure and format of the book clubs varied considerably. As other researchers have noted (Castellano et al. 2008), the design of the book clubs is likely to have an important effect on their success. While experimenting with different methods for implementing the book clubs, we tried a variety of models (e.g., solely online discussions, a mix of online and in-person discussions), finding that some worked better than others. We have identified several best practices for incorporating book club into sociology courses. Best practices are based largely on student feedback.

First, group sizes should be small, with four or five students being ideal. Although we experimented with larger groups (11–12 students) in one case, this did not work as well. All of the classes that the book clubs were implemented in were fairly small (under 45 students). However, we believe that the activity could be adapted for larger classes. One suggestion for larger classes would be to assign one book to multiple groups. For example, an instructor could have students choose between four books and then have three groups for each book, for a total of 12 groups. This would keep the course preparation comparable to smaller classes. It could also lend itself to new potential activities, such as having several groups with the same book engage in debates or peer review activities. As noted previously, although we focused on offering books that covered a variety of topics, instructors could also offer a range of perspectives pertaining to a debate or question, which could then allow for in-class debates drawing on evidence from the books.

Second, guided reading questions should be used to facilitate students’ understanding of the content of the books and give students an idea of particular concepts or events to pay attention to. Particularly within introductory-level courses, students may not have experience reading or engaging with social science research or long monographs. The guided reading questions can clarify difficult or new concepts and help students feel more confident that they understand the key points of the readings. Student surveys (see Table 2) suggest that students felt that the guided reading questions were an integral and important part of the book clubs.

Third, groups should meet multiple times, with different questions or objectives for each meeting. Although online forums were a good starting point for discussions, the book clubs were most successful when they also combined in-person discussions. Online discussions gave students the opportunity to prepare materials ahead of time and helped them develop their own positions. In-person discussions facilitated stronger connections between students and allowed them to teach each other. By helping students get to know each other, book clubs can have spinoff effects that go beyond facilitating an understanding of the books. In Jobs and Work, for example, the instructor noticed that many of her groups ended up working together beyond the book club settings (e.g., by studying together for exams). The book club groups also facilitated better in-class discussions, since students became very comfortable interacting with each other and asking questions.

Finally, instructors should integrate lessons and examples from the books into the class. This can be done through informal group discussions and by incorporating more formal strategies, such as charts. For student-led efforts, short oral presentations are best because they allow groups to share information about their books but do not require a lot of class time.

These recommendations are based on our own observations and on student feedback. For example, some students stated that they did not like the format of the online discussions. In the survey conducted in International Development (Fall 2009), the lowest average score was associated with the statement, “I enjoyed participating in the online ‘book club’ discussions” (see Table 1). As noted previously, some students found the follow-up questions to the discussion questions tedious or confusing. Other students felt that the online format was too impersonal. “I found it difficult to bring myself to argue with someone in an online discussion board. I didn’t want to make someone feel bad,” commented one student. Another student called for “some kind of integration with the group members and/or the book in general beyond the online discussion.”

It is for these reasons that we recommend a combination of online and in-person discussions. Based on the survey conducted in Jobs and Work, students had positive impressions of the book clubs in general and about the group size and discussion questions (see Table 2). For example, one student wrote that they enjoyed the book club discussions because they “made class different, so that it wasn’t the exact same thing every class.” The same student continued, “Also, the guided reading questions were very helpful in steering me in the right direction in terms of what I should have gotten out of the reading” (Jobs and Work, Fall 2014).

Students’ impressions of the group presentations were not as positive as their impressions of other aspects of the book clubs. In the survey conducted in Jobs and Work, scores for the questions about the presentations were lower than scores for the other questions. One student stated, “I felt like the individual group discussions were good; however, I didn’t feel like I gained anything from [group] presentations” (Jobs and Work, Fall 2014). Another student suggested that the presentations be integrated throughout the semester rather than leaving them until the end.

These “best practices” provide a useful starting point for instructors who want to implement book clubs in their own classes. Future research could further refine the list of best practices by testing specific elements of the design of the book clubs in an experimental way (e.g., by having students in one section do their group presentations at the end of the semester and having students in another section of the same course do their group presentations throughout the semester).

## Conclusions

As the examples in this paper show, book clubs can be integrated into a variety of different courses. In addition to the courses discussed here, book clubs could be easily integrated into courses on social problems, gender, race, family, and deviance, for example. Both scholarly books and popular nonfiction worked well for the book clubs. Although this article is based on book clubs implemented in relatively small classes at a large public university, one of the strengths of the book clubs is their flexibility and ability to engage students with wide-ranging interests and sociological fluency. Book clubs therefore could be used at a variety of institutions (e.g., community colleges, liberal arts colleges, or universities).

In general, students’ perceptions of the book clubs were favorable. This is demonstrated by surveys incorporating both quantitative and qualitative feedback. Many students expressed that the book club reinforced or illuminated new areas regarding the course materials. Book clubs were particularly successful at stimulating student engagement; for many students, they seemed to be among the most memorable activities that students participated in during the class. Book clubs also facilitated student learning. Students retained information from the book clubs and reported that the book clubs had helped them apply sociological concepts to real-world examples.

We conclude that book clubs are an effective pedagogical tool because of their flexibility and ability to foster student engagement and higher-level thinking. Future research should test the effectiveness of book clubs in comparison with other activities and assignments in sociology courses.

## Appendix

**Appendix:** Books and Topics Used. Books Used: International Development.

Title	Topic
*They Poured Fire on Us From the Sky: The Story of Three Lost Boys from Sudan* (Ajak, Deng, and Deng 2005)	Civil war in Sudan and refugees
*A Long Way Gone: Memoirs of a Boy Soldier* (Beah 2007)	Civil war in Sierra Leone
*Emergency Sex and Other Desperate Measures* (Cain, Postlewait, and Thomson 2004)	United Nations humanitarian aid in multiple contexts
*Kind Leopold’s Ghost: A Story of Greed, Terror, and Heroism in Colonial Africa* (Hochshild 1998)	Colonialism in the Congo
*Mountains beyond Mountains: The Quest of Dr. Paul Farmer, a Man Who Would Cure the World* (Kidder 2003)	Health and development in Haiti and Peru
*Three Cups of Tea: One Man’s Mission to Promote Peace . . . One School at a Time* (Mortenson and Relin 2006)	Education and peacemaking in Pakistan and Afghanistan
*Enrique’s Journey* (Nazario 2006)	Migration from Central America to the United States
*The Devil’s Highway: A True Story* (Urrea 2004)	Migration from Mexico to the United States
*Factory Girls: From Village to City in a Changing China* (Chang 2009)	Migrant workers in China
*Blue Clay People: Seasons on Africa’s Fragile Edge* (Powers 2008)	Aid work in Liberia
*Chasing Chaos: My Decade in and out of Humanitarian Aid* (Alexander 2013)	Aid work in Haiti, Sierra Leone, and Sudan
*Midnight in Mexico: A Reporter’s Journey through a Country’s Descent into Darkness* (Corchado 2013)	Drug trafficking in Mexico
*I Am Malala: The Girl Who Stood up for Education and Was Shot by the Taliban* (Yousafazi and Lamb 2013)	Education, human rights, and terrorism in Pakistan
Title	Topic
Books Used: Jobs and Work.	
*Fast Food, Fast Talk: Service Work and the Routinization of Everyday Life* (Leidner 1993)	Service work
*Gender Trials: Emotional Lives in Contemporary Law Firms* (Pierce 1996)	Professional work
*Inside Toyland: Working, Shopping, and Social Inequality* (Williams 2006)	Retail work
*Transnational Tortillas: Race, Gender, and Shop-floor Politics in Mexico and the United States* (Bank Muñoz 2008)	Globalization and factory work
*No More Invisible Man: Race and Gender in Men’s Work* (Wingfield 2013)	Professional work
*Code Green: Money-driven Hospitals and the Dismantling of Nursing* (Weinberg 2003)	Nursing
*Servants of Globalization: Women, Migration and Domestic Work* (Parreñas 2001)	Care work and globalization
*Race and the Invisible Hand: How White Networks Exclude Black Men from Blue-collar Jobs* (Royster 2003)	Manual labor and social networks
Title	Topic
Books Used: Principles of Sociology class.	
*Learning the Hard Way: Masculinity, Place, and the Gender Gap in Education* (Morris 2012)	Gender and education
*Nine Lives: Adolescent Masculinities, the Body, and Violence* (Messerschmidt 1999)	Adolescent male offenders
*The Managed Heart: Commercialization of Human Feeling* (Hochschild 1983)	Workplaces and emotion

## Notes

1. For International Development (Fall 2009), 31 out of 42 students participated (74 percent response rate). For Jobs and Work (Spring 2014), 14 out of 17 students participated in (82 percent response rate).

2. Geertsen (2003) defines higher-level thinking as a disciplined, systematic way of using the mind to confirm existing information (critical thinking) or search for new information (reflective thinking) using various degrees of abstraction.

3. The average score for the first question was 13.46 (SD = 1.99, n = 28). The question stated, “Rank the book club books from 1–6, with ‘1’ being the least alienating and ‘4’ being the most alienating. Give at least one justification for each book for its place in comparison to the other books.” The average score for the second question was 11.69 (SD = 3.20, n = 27). The question stated, “Choose two of the book club books (one of which can be your own). Using the books, describe how the sociological imagination comes into play. Discuss at least one public issue and one personal trouble for each book.” The average score for the third question was 13.80 (SD = 1.79, n = 42). The question stated, “Choose two of the following: race, class, gender, and ethnicity. Discuss at least one specific example for each topic from our course materials and how these characteristics can either advantage or disadvantage workers. For example, use a specific reading to discuss race and a specific book club book to discuss gender.”
